# Effect of Ivermectin on the Expression of P-Glycoprotein in Third-Stage Larvae of *Haemonchus contortus* Isolated from China

**DOI:** 10.3390/ani13111841

**Published:** 2023-06-01

**Authors:** Xiaoping Luo, Shuyi Wang, Ying Feng, Penglong Wang, Gaowa Gong, Tianlong Guo, Xingang Feng, Xiaoye Yang, Junyan Li

**Affiliations:** 1 Key Laboratory of Grass-Feeding Livestock Healthy Breeding and Livestock Product Quality Control, Inner Mongolia Academy of Agriculture and Animal Husbandry Sciences, Hohhot 010030, China; luoxpnmg@163.com (X.L.); baofengying08115@163.com (Y.F.); wangpenglong@cau.edu.cn (P.W.); gaowa2009c@163.com (G.G.); guotianlong831@163.com (T.G.); 2 College of Veterinary Medicine, Inner Mongolia Agricultural University, Hohhot 010018, China; 3 Shanghai Veterinary Research Institute, Chinese Academy of Agricultural Sciences, Shanghai 200241, China; xingangf62@aliyun.com; 4 Inner Mongolia Autonomous Region Comprehensive Center for Disease Control and Prevention, Hohhot 010031, China; shuyi1986721@163.com; 5 College of Veterinary Medicine, China Agricultural University, Beijing 100193, China

**Keywords:** *Haemonchus contortus*, ivermectin, P-glycoprotein, real-time fluorescence quantitative PCR

## Abstract

**Simple Summary:**

*Haemonchus contortus* is well known as the most harmful gastrointestinal nematode in ruminants, causing serious economic losses worldwide. The overuse of chemical insecticides (e.g., ivermectin) has caused gastrointestinal nematode resistance to develop and spread, resulting in failure in controlling gastrointestinal nematode spread. At the start of our study, four varieties of *H. contortus* were obtained from diverse regions of China. This study investigated the drug resistance of *H. contortus* isolates to ivermectin. Additionally, the changes in the *P-gp* gene transcription levels of third-stage larvae under ivermectin stress at different time points were measured. The results indicated that the YCHc-022 strain was sensitive to ivermectin, but the WSHc-001, WSHc-003, and CYHHc-136 strains displayed different degrees of resistance to ivermectin, in comparison with the ivermectin-sensitive *H. contortus* found in Australia. Results from the third-stage larvae of *H. contortus* in response to ivermectin stress revealed a relation between the *P-gp* genes and the drug resistance of *H. contortus* to ivermectin; however, it was not suitable for use as an international molecular marker. Our findings are beneficial for screening molecular markers of *H. contortus* resistance to ivermectin and offer a novel perspective for detecting the drug resistance of gastrointestinal nematodes.

**Abstract:**

*Haemonchus contortus* poses a severe hazard to the healthy development of the sheep industry and threatens the welfare of sheep. Ivermectin is the primary anthelmintic used for the prevention and treatment of *H. contortus* parasitism. However, the widespread and uncontrolled application of ivermectin has resulted in the development and spread of resistant strains of *H. contortus*. P-glycoprotein (P-gp) plays important roles in the pharmacology and toxicology of ivermectin, and changes in *P-gp* expression levels can be used to analyze the resistance of *H. contortus* to ivermectin. This study aimed to analyze the effects of ivermectin on *P-gp* expression in *H. contortus* L3 larvae isolated from China and to evaluate whether changes in *P-gp* expression levels can be used to analyze resistant *H. contortus* strains. In the absence of drug treatment, the ivermectin-resistant strains isolated in China showed increased expression of *P-gp*11 (*p* < 0.01) compared with sensitive strains from elsewhere, whereas the expressions of *P-gp*2 and *P-gp*9.1 were downregulated (*p* < 0.01). When the same strain was compared before and after drug treatment, obvious differences in expression were observed between the different strains. Ivermectin-induced *P-gp* expression was found to be very complex among the L3 larvae of different strains. In addition, it was confirmed that using *P-gp* to determine ivermectin resistance in *H. contortus* strains from different geographic environments can yield different results.

## 1. Introduction

Gastrointestinal nematode infections can seriously harm the health and welfare of sheep and, thus, can have a major impact on the development of the sheep industry [1,2]. Anthelmintic drugs, especially macrocyclic lactones based on ivermectin, have long played a major role in the control of these diseases. However, frequent and continuous medication usage, combined with a lack of evaluation of the effects of ivermectin after anthelmintic treatment, have led to increasing ivermectin resistance in sheep gastrointestinal nematodes worldwide, including in *Haemonchus contortus* [3,4,5,6,7,8]. According to Han et al., the infection rate of gastrointestinal nematodes (including *H. contortus*) in 10 grazing sheep in eastern Inner Mongolia was quite high, averaging 79.2% (from 45% to 100%), and the average infection intensity EPG was 1813 (from 0 to 32,400). In addition, the sheep displayed varying levels of resistance to avermectin, ivermectin, and albendazole [9]. A longitudinal study (from May 2013 to May 2017) was conducted and report that over 90% of eggs detected in the first month of investigation belonged to the species *H. contortus* and *Chabertia* sp. and that the gradual increase in the percentage of *H. contortus* eggs among all detected eggs during the study and the low cure rate of IVM mass treatment revealed the emergence of IVM resistance in *H. contortus* in Hubei Province, China [10]. The resistant *H. contortus* was also recorded in Brazil [11], the USA [12], South Africa [13], and the UK [14]. Despite attempts, no molecular marker has yet been identified to efficiently, accurately, and quickly assess the resistance of *H. contortus* to ivermectin.

Ivermectin is a macrolide anthelmintic and a ligand-gated ion channel agonist [15,16]. It primarily acts on glutamate and γ-aminobutyric-acid-gated chloride channels through irreversible binding with channel subunits. This induces an increase in chloride ion permeability, which in turn increases the intracellular chloride ion concentration, thus polarizing the parasite’s neuromuscular system and triggering the inhibition of pharyngeal pumping and feeding. This results in neuromuscular paralysis and decreases reproductive function, which reduce the ability of the parasite to remain within a host and to feed on the host, and limit the parasite’s level of eating, finally resulting in detachment from the host or in death due to lack of nutrition [17,18]. Despite extensive research into the insecticidal effects of ivermectin on parasites, the resistance mechanism of parasites to ivermectin remains unclear.

Ivermectin resistance in *H. contortus* is associated with the mechanism of intracellular drug efflux; nematodes use this mechanism to clear intracellular toxins and foreign substances. This mainly involves changes in the expressions of members of the ATP-binding cassette (ABC) transporter superfamily, including P-glycoprotein (*P-gp*) and multidrug-resistance-associated proteins (Mrps) [19,20,21]. The ABC transporter gene family is the largest transporter gene family in the metazoa, and its primary purpose is to transport toxic substances out of cells. This gene family is divided into eight subfamilies (ABCA-ABCH). Research has revealed that ABC transporters are linked to pesticide resistance, with the ABCB (P-gp) subfamily facilitating the efflux of pesticides or their metabolites. Many studies have indicated that the expression of the *P-gp* gene is in-creased or that the ABC transporter inhibitor verapamil has a synergistic effect in resistant strains. Reports have shown that the expression of ABC transporter genes is upregulated after exposure to various pesticides in numerous parasites. Many studies have shown that P-gp plays an important role in the pharmacology and toxicology of ivermectin; it can control the distribution of ivermectin in all tissues of an organism. In mammals, P-gp in intestinal epithelial cells can excrete ivermectin into the intestine, causing the ivermectin concentration in the intestine to sharply increase [22]. P-gp also exists in the intestines of nematodes [23]. Therefore, it has been speculated that *H. contortus* can also excrete ivermectin, thereby reducing its effectiveness and resulting in drug resistance. Based on this, changes in the expression level of *P-gp* have been used to analyze its relationship with ivermectin-resistant strains of *H. contortus* [24]. The connection between *P-gp* gene expression and *H. contortus* resistance to ivermectin is known, yet it remains uncertain if this is a universal marker for drug resistance.

Considering the question of whether changes in the expression levels of *P-gp* can also be used to analyze ivermectin resistance in *H. contortus* isolated in China, we conducted a larval development experiment to measure the resistance of *H. contortus* isolates from different areas to ivermectin. Additionally, we examined the transcription level of *P-gp* genes in the third-stage larvae of *H. contortus* under ivermectin stress to determine if P-gps can be used as a molecular marker of ivermectin resistance in *H. contortus*.

## 2. Materials and Methods

### 2.1. Ethical Declaration

This study was approved by the Animal Ethics Committee of the Shanghai Veterinary Research Institute, Chinese Academy of Agricultural Sciences (SHVRI-SZ-20200723-01). All sheep were handled in strict accordance with good animal practices according to the Animal Ethics Procedures and Guidelines of the People’s Republic of China.

### 2.2. Parasites

The sensitive *H. contortus* strain (Hc-S) originated from Australia; it has been maintained in sheep at the Inner Mongolia Academy of Agriculture and Animal Husbandry Sciences since 2015. Strains WSHc-001, WSHc-003, CYHHc-136, and YCHc-022 were isolated from North China in 2019 (Table 1) and have since been maintained in sheep. Previous fecal egg count reduction tests showed that the WSHc-001, WSHc-003, and CYHHc-136 strains were resistant to 1.2 mg·kg^−1^ ivermectin, whereas the YCHc-022 strain was sensitive to 0.2 mg·kg^−1^ ivermectin [25].

To obtain the eggs required for the larval development inhibition test, fecal samples were collected from sheep rectums and placed in a mortar, an appropriate amount of saturated saline solution was added, and the mixture were ground. Next, 1000 mL of saturated salt water was added, mixed well, and sequentially filtered through 40- and 100-mesh filters. A total of 200 mL of saturated sucrose in water was then added to the filtrate, mixed well, and poured into a large plate. Saturated salt water was added until the liquid level was slightly higher than the mouth of the plate. The plate was then covered with a hard plastic film and allowed to stand for 15 min. Next, the hard plastic film covering the plate was gently lifted, the eggs on the surface of the film were flushed into a beaker with deionized water, and the eggs were rinsed using a centrifugal machine centrifuging them 3–5 times at 3000 rpm. Finally, microscopic examinations of the eggs were performed. After confirming that no large particles of impurities were present, the eggs were counted and diluted into a solution with a concentration of approximately 5000 eggs·mL^−1^.

To obtain the L3 larvae needed for the experiments, fresh feces were collected in plastic bags and brought back to the laboratory, and samples were placed on an enamel tray. After culturing at 27 °C for seven days, L3 larvae were collected using the Baermann technique. The collected *H. contortus* L3 larvae were washed repeatedly using deionized water to remove impurities and were then examined under a microscope to confirm that there was no contamination from other nematodes.

### 2.3. Larval Development Assay

To determine the level of drug resistance of *H. contortus* isolates from various regions to ivermectin, we conducted a larval development experiment, in which the experimental group was exposed to different concentrations of ivermectin. Cultures were conducted in 24-well culture plates. Twelve ivermectin concentrations were set up for each group (272, 136, 68, 34, 17, 8.5, 4.25, 2.12, 1.06, 0.53, 0.26, and 0.13 ng·mL^−1^), along with one negative control group (0.5% dimethyl sulfoxide; DMSO) and one positive control group (1.09 mg·mL^−1^ ivermectin; initial testing revealed that 1.09 mg·mL^−1^ ivermectin successfully eradicated the third-stage larvae of *H. contortus* resistant strains, achieving a mortality rate of 100%, but has not been formally published yet). The total volume in each well was 300 μL, which included the 20 μL egg suspension, 30 μL ivermectin working solution of different concentrations, 50 μL NCTC-109 medium solution, 1 μL water-soluble amphotericin B solution (500 μg·mL^−1^), and 199 μL deionized water. The experiment was replicated three times independently, and each of these replications was conducted with two technical repeats.

After setting up the cultures, the culture plate was placed in a biochemical incubator at 27 °C for seven days, and 5 μL of Lugol’s solution was added to each well. The numbers of unhatched eggs, L3 larvae, and larvae that had not yet developed to L3 were counted using an inverted microscope. The log(agonist) vs. response-variable slope (four parameters) method in GraphPad Prism 6 software was used to calculate the half maximal effective concentration (EC_50_) and R^2^ values, and dose-response curves were plotted. The resistance ratio (RR) was calculated based on EC_50_, as shown below; a RR > 2 was considered to represent resistance:

RR = EC_50_ of the tested strain/EC_50_ of the sensitive strain

### 2.4. RNA Extraction and Reverse Transcription

To detect the changes in *P-gp* gene transcription levels in the third-stage larvae of *H. contortus* due to ivermectin stress, the third-stage larvae were subjected to treatment. L3 larvae of each strain of *H. contortus* were placed in a 6-well plates. Each strain was placed into six wells and divided into three groups, with approximately 50,000 larvae in each well. In addition, a 50% 1640 culture medium was added to maintain a liquid surface height of 2–3 mm.

For each strain, group 1 was not given any treatment, and the bodies of the larvae were directly collected and stored in liquid nitrogen. For groups 2 and 3, an ivermectin solution was added to a final concentration of 1 μg·mL^−1^ and wells were cultured in a 32 °C incubator. Group 2 was cultured for 3 h and then removed from the incubator. The culture medium was then discarded, and the larvae were rinsed three times with deionized water and then collected and stored in liquid nitrogen. Group 3 was cultured for 6 h and then removed from the incubator, rinsed, and stored in liquid nitrogen.

The collected L3 larvae from the ivermectin-treated and control groups were then rapidly frozen in liquid nitrogen and stored at −80 °C for RNA extraction. Total RNA was extracted from the whole L3 larvae samples via homogenization using an automill of OSE-Y20 TGrinder (Tiangen Biotech Co., Ltd., Beijing, China) and was added to a TRIzol Reagent (Thermo Fisher Scientific, Shanghai, China). RNA extraction was performed following the manufacturer’s protocol. To check the RNA quantity, the absorbance at 260 nm and the absorbance ratio of OD260/280 were measured with a Nanovue UV–Vis spectrophotometer (GE Healthcare, Fairfeld, CT, USA). Subsequently, the reverse transcription was carried out using TransScript One-step gDNA Remover and cDNA Synthesis SuperMix (TransGen Biotech, Beijing, China), and the synthesized cDNAs were stored at −20 °C. Finally, the complementary deoxyribonucleic acid (cDNA) of each *H. contortus* strain was obtained, both with (at 3 and 6 h) and without treatment.

### 2.5. Quantitative Real-Time PCR

The transcription levels of the *P-gp* genes in different strains and after the induction of ivermectin were determined via qPCR using a StepOnePlus™ Real-Time PCR system (Thermo Fisher Scientific, Waltham, MA, USA). The volume of reaction mixtures was 20 μL, containing 10 μL of the TransStart Tip Green qPCR SuperMix (TransGen Biotech, China), 7.2 μL of ddH2O, 0.4 μL of a forward primer (0.2 μM), 0.4 μL of a reverse primer (0.2 μM), and 2 μL of cDNA. The amplification protocol for qRT-PCR was initial denaturation at 95 °C for 10 min, followed by 40 cycles of 95 °C for 15 s and 60 °C for 1 min. The fluorescence signal was measured at the end of each extension step at 60 °C. To ensure consistency and specificity of the amplified products, we also performed melt curve analyses (from 60 to 95 °C). Three biological replicates were conducted for an qRT-PCR experiment. The actin was used as an internal reference gene for *H. contortus*. The *P-gp* fluorescence-based quantitative PCR primers and conditions reported by Williamson et al. [24] were used. The 2^−ΔΔCT^ method was used to calculate the mRNA expressions of the target genes. GraphPad Prism 6 software was used for statistical analysis of the data.

### 2.6. Statistical Analysis

Data analysis was performed using GraphPad Prism version 6.0.0 for Windows (GraphPad Software, San Diego, CA, USA), and the results were represented as the mean ± SE with biological replicates. Student’s *t*-test was employed to compare the discrepancies between two samples in the larval development assay. An independent sample *t*-test was utilized to evaluate the relative expression levels of the *P-gp* gene after induction by ivermectin at different time points and *H. contortus* L3 of different isolates, with *p* < 0.05 as the criterion for statistical significance.

## 3. Results

### 3.1. Resistance Status of the Four H. contortus Isolates

**Figure 1:**
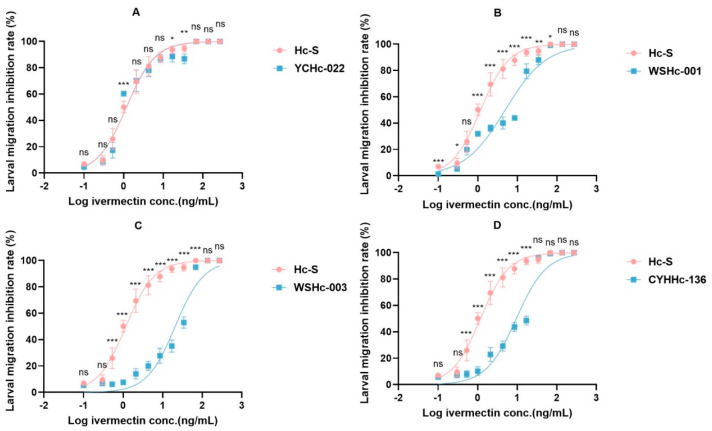
Detection result of IVM resistance of different isolates of *H. contortus.* The ivermectin resistance of different isolates of *H. contortus* was evaluated via a larval developmental inhibition assay. The international strain of *H. contortus* (Hc-S), which was sensitive to ivermectin, was used as a reference, and strains YCHc-022 (**A**), WSHc-001 (**B**), WSHc-003 (**C**), and CYHHc-136 (**D**) were sensitive to ivermectin. The significance level was set at * *p* < 0.05, ** *p* < 0.01, and *** *p* < 0.001 and “ns” represent non-significant compared with the control (blank).

The ivermectin resistances of the different *H. contortus* isolates were tested using a larval development inhibition test. A larval development inhibition test showed that the different ivermectin resistances were caused by the *H. contortus* isolates. The ivermectin sensitivity of WSHc-001, WSHc-003, and CYHHc-136 were researched based on *H. contortus* strains from Australia, and all met the condition of RR < 2, indicating that they were resistant to ivermectin. For the YCHc-022 strain, however, RR = 1.006, indicating that it was sensitive to ivermectin (Table 2). The dose–response curves of ivermectin also reflected the same result (Figure 1).

### 3.2. Expression of P-gp for Each Isolate before Drug Stimulation Compared with Hc-S

**Figure 2:**
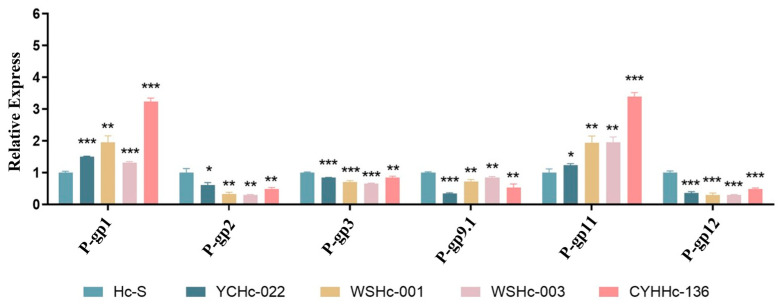
Expression of P-gps of each isolate before drug stimulation was compared with Hc-S. The significance level was set at * *p* < 0.05, ** *p* < 0.01, and *** *p* < 0.001. Data are representative of three independent experiments.

In the absence of ivermectin stimulation, the *P-gp* expressions of the four isolated strains were significantly different from the susceptible strains from Australia (Hc-S). Specifically, the expressions of *P-gp*1 and *P-gp*11 were higher in the isolates than in the susceptible strains from Australia, whereas the expressions of *P-gp*2, *P-gp*3, *P-gp*9.1.1, and *P-gp*12 were significantly downregulated (Figure 2). A comparison of the isolated resistant and sensitive strains revealed that *P-gp*2 and *P-gp*11 exhibited small changes in the sensitive strains, whereas *P-gp*9.1.1 exhibited a relatively large change.

### 3.3. P-gp Expressions of the Same Isolate before and after Drug Stimulation

Analyzing the *P-gp* expressions in the sensitive strains from Australia before and after treatment showed that the expressions of *P-gp*3, *P-gp*9.1, and *P-gp*12 decreased significantly after treatment. The expression levels of *P-gp*2 and *P-gp*11 decreased slowly after treatment, whereas that of *P-gp*1 increased briefly and then decreased.

For the susceptible strain YCHc-022 that was isolated in China, the expressions of *P-gp*1, *P-gp*3, *P-gp*9.1.1, and *P-gp*11 decreased significantly after treatment, that of *P-gp*12 increased significantly, and that of *P-gp*2 showed no significantly change.

**Figure 3:**
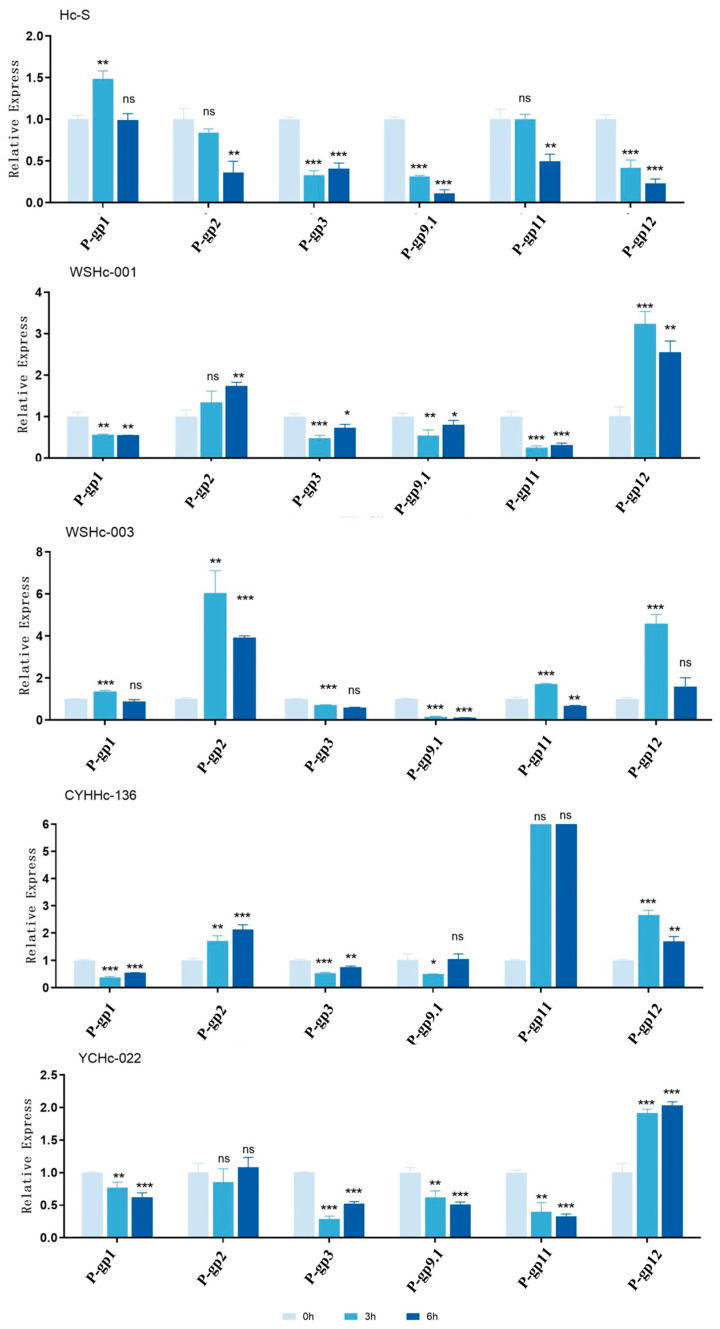
Expression of P-gps of each isolate before and after drug stimulation. Expression of P-gps of each isolate was detected both with (at 3 and 6 h) and without treatment. The significance level was set at * *p* < 0.05, ** *p* < 0.01, and *** *p* < 0.001, and “ns” represents non-significant compared with the control (blank). Data are representative of three independent experiments.

For the different resistant isolates, the expression levels of *P-gp*3 and *P-gp*9.1 decreased significantly after treatment, those of *P-gp*2 and *P-gp*12 increased significantly, and those-gp1 and *P-gp*11 showed no completely consistent significant changes (Figure 3).

## 4. Discussion

*H. contortus* is one of the most harmful parasites in sheep. Until non-chemical parasite control technologies (such as vaccines and biological control) mature, chemical anthelmintics will play a major role in the prevention and control of *H. contortus* infections. Parasitic resistance to anthelmintics is the primary problem plaguing chemical anthelmintic treatment. Despite the abundance of research on parasite resistance, the molecular mechanisms behind parasite resistance are still poorly understood, and there are several aspects that warrant further investigation [26]. With the infection rate of *H. contortus* being high in China and its strong resistance to ivermectin, it is imperative to find a reliable, fast, and accurate method to detect its drug resistance level. Additionally, understanding its drug resistance mechanism is essential in order to create more effective prevention and control strategies for *H. contortus*. *H. contortus* has numerous sources and a wide-distribution, rich genetic diversity, and it is easy to culture and transport. It has been the target of abundant basic research and has a close genetic relationship with the model organism *Caenorhabditis elegans*. Thus, it can itself serve as a model organism for studying the resistance of parasitic worms to anthelmintics (e.g., ivermectin) [27,28]. Investigating gastrointestinal nematodes is mainly concentrated on *H. contortus*, particularly on prevention, control, and the drug resistance mechanism.

Research into existing *H. contortus* strains has shown that the strains used within or between different laboratories worldwide have very high genetic diversity [29,30,31]. Thus, given that strains from different countries may not fully represent the genetic characteristics of strains from China, and given the lack of strains related to the drug resistance of *H. contortus* in China, conventional methods were used in this study to isolate ivermectin-resistant and ivermectin-sensitive *H. contortus* strains in China. Among the four strains isolated, three were resistant to a six-fold dose of ivermectin (1.2 mg·kg^−1^), and only one was sensitive to the recommended dose of ivermectin (0.2 mg·kg^−1^) [25]. The results of this study are essential for the development of ivermectin-resistant strains of *H. contortus* in China and will also serve as a fundamental source of information for the study of the drug resistance mechanism of *H. contortus* in the future.

The Fecal egg reduction test is considered the best way to determine if gastrointestinal nematodes are resistant to insecticides, but it requires live animals, takes a long time, and is expensive. Larval development inhibition tests have been widely used as a routine technique for in vitro analysis of drug resistance in *H. contortus* [32,33,34,35]. Here, this technique was used to measure the level of ivermectin resistance in different *H. contortus* isolates in vitro. The RRs of the four isolates were found to differ; the RRs of the three resistant strains were 5.82, 11.26, and 26.00, whereas that of the susceptible strain was only 0.89. The results of the larval development inhibition test are in agreement with the fecal egg reduction test, indicating that this test is reliable for determining the resistance of gastrointestinal nematodes to anthelmintics in vitro. This implies that the sensitive strain also has the capacity to serve as a sensitive standard strain of Ivermectin for *H. contortus* in China.

P-gp is a member of the ABC family of efflux pumps. It can remove lipophilic exogenous compounds from cells and is considered to be associated with resistance to anthelmintics [36]. Blackhall et al. [37] revealed *P-gp* to be associated with the resistance of *H. contortus* to macrolide drugs. Using *C. elegans P-gp* as a reference, Williamson et al. [38] identified nine related P-gps in *H. contortus*, namely *pgp*-1, *pgp*-2, *pgp*-3, *pgp*-4, pgp-9, *pgp*-10, *pgp*-11, *pgp*-12, and *pgp*-14. Furthermore, they analyzed the expressions of these nine P-gps in ivermectin-resistant and ivermectin-sensitive *H. contortus*, finding no statistically significant differences in expression levels between the different strains. Raza et al. [39] showed that the expression levels of at least three *P-gp* genes in ivermectin-resistant *H. contortus* strains were higher than in ivermectin-sensitive strains. They also identified that five *P-gp* genes were highly expressed in *H. contortus* after ivermectin stimulation. Consequently, it is essential to analyze the *P-gp* gene expression level in distinct *H. contortus* strains that are resistant to ivermectin, so as to more accurately comprehend the drug resistance mechanism in different regions.

In the present study, the infectious L3 larvae of *H. contortus* from different isolated strains were used as the research subjects. In the absence of ivermectin stimulation, the expressions of *P-gp*1 and *P-gp*11 increased significantly in the three isolated drug-resistant strains, compared with the Hc-S sensitive strain from Australia, whereas the expressions of *P-gp*2, *P-gp*3, *P-gp*9.1.1, and *P-gp*12 were significantly downregulated (Figure 2). Significant differences were also observed in the changes in expressions between strains from Australian and Chinese isolated strains, when compared before and after drug treatment. This was particularly true for *P-gp*12, the expression of which increased significantly after drug stimulation in strains isolated in China and the expression levels of which decreased significantly in strains from Australia (Figure 3). However, a change in expression level is not the only factor that determines drug resistance in drug-resistant strains from different sources; resistance mechanisms also depend on different regions and specific conditions [40].

## 5. Conclusions

In summary, three ivermectin-resistant strains of *Haemonchus contortus* and one ivermectin-sensitive strain isolated from North China, as well as one ivermectin-sensitive strain isolated from Australia, were investigated in the present study. A gene expression analysis of *P-gp* in the different strains showed that there may be some relationship between *P-gp* and ivermectin resistance in *H. contortus*. In addition, the changes in its expression levels were found not to be a determinative factor of drug resistance in *H. contortus* strains from different origins. Therefore, *P-gp* cannot be used as a drug resistance marker molecule for developing a uniform standard applicable across different regions, especially regarding countries with different geographic environments and anthelmintic treatment patterns. It is clear that there are differences in the mechanisms of ivermectin resistance in different *H. contortus* strains. Studies should be conducted in different regions to screen for molecular markers suitable for the detection of ivermectin resistance in *H. contortus* in China.

## Figures and Tables

**Table 1:** Source of all isolates and characteristics of resistance to ivermectin.

Isolate	Degree of Resistance	Source of Isolate
Hc-S	0.2 mg/kg	Australian
WSHc-001	1.2 mg/kg	Northern China, N 39°5′52″ E 109°14′27″
WSHc-003	1.2 mg/kg	Northern China, N 39°8′54″ E 109°18′8″
CYHHc-136	1.2 mg/kg	Northern China, N 41°27′47″ E 113°18′9″
YCHc-022	0.2 mg/kg	Northern China, N 37°76′29″ E 112°83′61″

**Table 2:** EC_50_ and anthelmintics resistance ratio of different isolates of *H. contortus*.

Isolate	EC_50_ ng/mL	RR	R^2^
Hc-S	1.171	-	0.977
YCHc-022	1.178	1.006	0.946
WSHc-001	4.912	4.195	0.948
WSHc-003	20.56	17.558	0.948
CYHHc-136	9.41	8.036	0.952

## Data Availability

The data generated or analyzed during this study are included in this published article.

## Abbreviations

ABC	ATP-binding cassette
ATP	adenosine tri-phosphate
cDNA	complementary deoxyribonucleic acid
DMSO	dimethylsulfoxide
EC_50_	half maximal effective concentration
Hc-S	*Haemonchus contortus* strain
mRNA	messenger RNA
Mrps	multidrug resistance-associated proteins
PCR	polymerase chain reaction
P-gp	P-glycoprotein
RNA	ribonucleic acid
RR	resistance ratio
